# The impact of mulberry leaf extract at three different levels on reducing the glycemic index of white bread

**DOI:** 10.1371/journal.pone.0288911

**Published:** 2023-08-10

**Authors:** Fangli Ding, Qing Wang, Chen Xie, Meng Wang, Lu Zhang, Ming Gao, Zongling Yang, Jianrui Ma, Xiaodong Shi, Wei Chen, Shenglin Duan, Peng Yuan, Yali Li, Xishan Ma, Yimin Wu, Jia Liu, Xiaowen Feng, Qingli Cheng, Zichun Wang, Xuyan Li, Jingmei Huang

**Affiliations:** 1 Beijing key laboratory of the Innovative Development of Functional Staple and the Nutritional Intervention for Chronic Disease, China National Research Institute of Food and Fermentation Industries, Beijing, People’s Republic of China; 2 Institute of Medicinal Plant Development, Chinese Academy of Medical Science, Peking Union Medical College, Beijing, People’s Republic of China; 3 Department of Clinical Nutrition, Department of Health Medicine, Peking Union Medical College Hospital, Chinese Academy of Medical Sciences and Peking Union Medical College, Beijing, People’s Republic of China; 4 Beijing Engineering Research Center of Protein and Functional Peptides, China National Research Institute of Food and Fermentation Industries, Beijing, People’s Republic of China; 5 Beijing Key Laboratory of Forest Food Processing and Safety, College of Biological Sciences and Biotechnology, Beijing Forestry University, Beijing, People’s Republic of China; 6 College of Life Science and Food Engineering, Hebei University of Engineering, Handan, Hebei, People’s Republic of China; University of Milan, ITALY

## Abstract

In this study, the influences of mulberry leaf extract (MLE) addition on the physicochemical properties including the specific volume, texture and sensory features of white bread (WB) were evaluated by the sensory analysis technology. A double-blind, randomised, repeat-measure design was used to study the impact of MLE addition on the postprandial blood glucose response as well as the satiety index of WB. Results showed that the addition of MLE showed no significant effects on the physicochemical properties of WB except for the slight changes of color and bitterness. The addition of MLE significantly reduced the total blood glucose rise after ingestion of WB over 120 minutes, and reduced the GI value of WB in a dose-effect relationship. When the concentration of MLE reached 1.5 g per 100 g available carbohydrate, the GI value of WB could be reduced from 77 to 43. This study provides important information in terms of the appropriateness of MLE when added to more complex real food, the dose-dependent relationship could supply a reference for the application of MLE.

## Introduction

Diabetes mellitus (DM) has been considered one of the major public health challenges worldwide [1]. It was estimated that the global number of total DM-related health expenditures in 2021 was 966 billion, which had already doubled the amount compared with a decade ago. It is of great importance to control the growing public health burden and to find more economically efficient lifestyle intervention ways. The consumption of a low glycemic index (GI) diet has been proven to be one of the practical approaches to preventing the further progression of impaired blood glucose levels [2]. GI is a calculated number that indicates the ability of a certain carbohydrate-containing food to increase the postprandial blood glucose level compared with a reference food (generally glucose). It is a relative index describing the digestive characteristic of carbohydrates in certain foods, where food with GI ≤ 55 is classified as a low GI food. Studies have illustrated that the low GI diet was only been globally considered as a practical eating pattern for postprandial blood glucose management and DM prevention, but also was recommended as an overall healthier dietary pattern for the public [3]. Therefore, investigating ready-to-eat low GI foods that possess lower postprandial glycemic response would be of great importance.

To formulate low GI foods, besides using slowly digestible cereals and beans, adding natural ingredients with hypoglycemic effects is also a very effective way [4]. Over past decades, a certain number of medicinal plant phytochemicals have been considered as α-glucosidase inhibitors, and thus act as antidiabetic drugs such as the *Garcinia mangostana* (flavonoids), *Luculia pinciana* (terpenoids), *Adhatoda vasica Nees* (alkaloids) and *Morus alba* L. (iminosugar) [5]. Some studies in recent years have also proved that some functional components, including polyphenols, alkaloids, and polysaccharides, that widely exist in natural plants might be useful when developing low GI foods [6]. These plant extracts such as buckwheat extract, mulberry leaf extract (MLE), and grape seed extract could inhibit the digestion of carbohydrates by inhibiting digestive enzyme activities [7,8], and among them, MLE is extracted from the leaves of *Morus alba*, and contains various bioactive components such as alkaloids and flavonoids. 1-deoxynojirimycin (DNJ), one of the alkaloid components in MLE, is a D-glucose analog that competitively inhibits the enzymes participating in carbohydrates metabolisms, such as α-glycosidase and amylase [9]. Therefore, MLE may have a strong inhibitory effect on food digestion and has a broader prospect in the development of low GI products [10]. Despite the excellent a-glucosidase inhibitory activity of 1-deoxynojirimycin (DNJ), *in vitro*, its efficacy *in vivo* was only moderate. A recent study showed that an addition of MLE (equivalent to 7.5 mg of DNJ) to 50 g of different commonly simple carbohydrates (maltose, sucrose, maltodextrin, and glucose) has been found to show the reduction of their GI values by 53.11%, 33.51%, 31.00%, and 8.12%, respectively [10]. In another study, the addition of MLE containing 5% DNJ to 50 g maltodextrin at three different levels had been found to have a dose-dependent trend in reducing the total postprandial blood glucose rise of the maltodextrin [11]. However, the effectiveness of MLE in lowering the postprandial blood glucose levels of common complex foods has not been confirmed by human studies.

It is known that the DNJ content of mulberry leaves is usually influenced by the extraction processing step, and reduced after the prolonged high-temperature treatment of roasting [12]. In the present study, bakery production involves processes of mixing, fermenting, baking, etc., whether MLE could still possess the inhibition effect towards digestive enzymes under such circumstances remains unknown. Meanwhile, whether the addition of MLE towards a complex food would yield any characteristic changes to the food, in this study the WB, is yet to be determined. In addition, there is still a lack of evidence on the glycemic lowering effect and satiety changes of MLE when being added to real food. With the previous evidence, we hypothesized that adding MLE into foods was a determined way to reduce their GI value. Therefore, the influence of MLE on the WB properties was systematically evaluated in terms of characteristics alterations, *in vitro* digestion characteristics, *in vivo* glycemic lowering effect, as well as satiety.

## Materials and methods

### Ethical statement

The ethical approval for the study was obtained at the Chinese Academy of Medical Sciences and Beijing Union Medical College Hospital Ethics Review Committee (protocol code: HS-1763, first issued: November 27, 2018, latest updated on October 9, 2023). The written informed consent was obtained from all subjects involved in the study.

### Materials and sample preparation

In the current study, Breads for this study all shared the same basic formulation and a standardized secondary fermentation baking process (S1 Fig in S1 File). MLE (Sangduoan^®^, batch number ML20191218, Botanic Century (Beijing) Co. Ltd.) used in the bread was a food-grade commercially available aqueous extract of the leaves of *Morus alba* Linn that contained 1% DNJ in the local market. During the WB preparation process, MLE was firstly dissolved in water and then mixed with other ingredients to obtain the dough with a desirable texture. Immediately, the dough would go through two rounds of fermentation, at a temperature at 30°C for 60 min with 90% relative humidity per round. Then, the fermented dough could typically be baked for about 12 min at 175°C and the WBs were ready to test after cooling down to room temperature.

### Dough preparation

The available carbohydrate (AC) amount of the WB was tested by China’s national food standards GB 5009.9–2016 and GB 5009.8–2016, and the result was 53.29 g/100 g. In this study, based on previous research [10,11], the three levels for detecting the dose-depend trend of MLE glycemic lowering effect were set at 0.75 g (contained 7.5 mg DNJ, MLE1), 1.25 g (contained 12.5 mg DNJ, MLE2) and 1.5 g (contained 15 mg DNJ, MLE3) of per 100 g AC amount of the WB. Anhydrous glucose (Yi Nuo BioTech, Zhejiang, China) was used as the reference food for each GI determination test of this study.

### Characteristics determinations of white bread

#### Chromatic aberration

The color of the breads was analyzed by a spectrophotometer SE 6000 (Nippon Denshoku Industries Co., Ltd.). Data were reported in the form of CIE L*, a*, and b* color space.

Here, L* represents lightness. A positive a* represents redness, and a negative value symbolizes greenness. Positive and negative b* signifies yellowness and blueness, respectively [13].

For each sample, three spots were randomly selected and analyzed. CIE whiteness index was then calculated by Eq 1

$$\sqrt{{\left(100-\text{L}*\right)}^{2}+{\text{a}}^{*2}+{\text{b}}^{*2}}$$ (1)

#### Specific volume analysis

Breads were weighed and the loaf volume was measured using a seed displacement method [14] at the storage of 0 h. The specific volume of bread was calculated in the following Eq 2.

SV=V/m (2)

where: SV is the specific volume of bread (cm^3^/g), V is the volume of bread (cm^3^) and m is the mass of the bread (g). All measurements were quantified in at least in triplicates.

### Textural comparison

#### Textural properties

(2×2×2 cm^3^ were measured using a TA.XT Plus Texture Analyser (Stable Micro Systems Ltd, Surrey, UK). Texture profile analysis (TPA) was applied following the previous approach [15]. Hardness, cohesiveness, and chewiness were recorded.

#### Electronic tongue sensory comparison

Electronic tongue sensory analysis was conducted for each WB separately using an Insent Taste Sensing System TS-5000Z (Intelligent Sensor Technologies, Inc., Kanagawa-Pref., Japan) [16]. The detecting sensors used in this study were: CT0 (saltiness), AAE (umami), CA0 (sourness), C00 (bitterness and aftertaste-bitterness), AE1(astringency and aftertaste-astringency), GL1(sweetness). For each measurement, the reference value (Vr) was first recorded for the sensor in the reference solution (0.3 mM L-(+)-tartaric acid and 30 mM KCl), followed by a measurement of a sample (Vs) in the sample solution (35 mL). After sample measurement, the sensor was shortly washed with the reference solution and the reference value (Vr’) was recorded. Finally, the sensor was thoroughly cleaned in the alcohol solution (100 mM HCl in 30% ethanol for negatively charged membrane; 10 mM KOH and 100 mM KCl in 30% ethanol for positively charged membrane) before proceeding to the next sample. The difference Vs−Vr represents the initial taste while Vr’−Vr represents the aftertaste.

### *In vitro* studies

#### DNJ content determination

DNJ content in the WBs was determined according to the method which has been introduced in detail by Wulandari et al.[17], but only with minor modifications, Firstly, the DNJ was extracted from WBs with the assistance of ultrasonic (250W, 60°C, solid-liquid ratio 1:4, extraction solvent: 70% ethanol), Then the DNJ derivatization was conducted with borate solution (0.4 mol/L, pH = 8.5) and FMOC-Cl acetonitrile solution (5 mmol/L), and glycine solution (0.1 mol/L) was used to neutralize the remaining unreacted derivative reagents. The final analysis was performed by ultra-high liquid chromatography (UPLC) (ACQUITY UPLC H-Class, Shanghai, China) on a silica C18 column (4.6×250 mm, 5 μm) with a mobile phase system of 40% acetonitrile (40% solvent A) and 1% acetic acid (v/v, 60%, solvent B). The elution was carried out in isocratic mode with a flow rate of 0.3 mL/min with a total running time of 4 min at 30°C with the VWD detector set at 256 nm. All chromatographically pure chemical reagents used for UPLC analysis were purchased from Sinopharm Chemical Reagent Beijing Co., Ltd. (Beijing, China) and the purified water from Hangzhou Wahaha Group Co., Ltd. (Hangzhou, China).

#### *In vitro* digestibility assay

To explore the effect of MLE on the *in vitro* digestibility properties of WB, the hydrolysis rate of bread was determined based on the method of Brodkorb et al. [18] and Ding et al. [19] with moderate modifications, where all analytical grade chemical reagents for the assay were purchased from Sinopharm Chemical Reagent Beijing Co., Ltd. (Beijing, China).

The breadcrumb was freeze-dried and sieved through an 80-mesh sieve. The compositions of the simulated oral fluid, gastric juice, and small intestinal juice were shown in S1 Table in S1 File, where α-amylase (5 U/mg), pepsin (250 U/mg), pancreatin (250 U/mg) were all purchased from Sigma-Aldrich Chemical (MO, USA). In addition, the pH of the simulated digestive juices was adjusted with 1 mol/L HCl or 1 mol/L NaOH. The *in vitro* digestion of all bread samples was conducted as follows:

150 mg bread crumb powder was incubated in a drug dissolution apparatus (RC-806, Tianjin, China) at 150 rpm at 37°C, which consisted of the following incubations of 150 mg of oral fluid (digestion for 2 min), 10 mL of gastric juice (incubation for 1 h), and 10 mL of intestinal juice (incubated for 3 h). Aliquots (0.5 mL) were taken out and inactivated by adding 4.5 mL absolute ethanol at the small intestine stage (0, 20, 40, 60, 90, 120, 150, 180 min), the samples were centrifuged at 5000 rpm, 4°C for 5 min to obtain the supernatant. After that, the concentration of reducing sugars in the digestive solution was determined by the 3,5-dinitro salicylic acid method, which was finally converted to starch digestibility. The final digestion curve was plotted as starch digestibility versus time.

The calculated hydrolysis index (HI) was obtained by dividing the area under the curve (AUC) of the sample by the AUC obtained for WB. The expected glycemic index (eGI) was calculated using Eq (3) described by Granfeldt, et al.[20].


eGI=0.862HI+8.198 (3)

### *In vivo* GI study

#### Study design

According to the ISO standard: ISO 26642:2010, four standard GI determination tests were conducted to obtain the GI values of MLE WB and WB. The tests were conducted by the Beijing Key Laboratory of the Innovative Development of Functional Staple and the Nutritional Intervention for Chronic Disease (one of the accredited GI test labs of the Glycemic Index Foundation, Australia). The study was registered in the Chinese Clinical Trial Registry (Registration number: ChiCTR2100044474). The ethical approval for the study was obtained at the Chinese Academy of Medical Sciences and Beijing Union Medical College Hospital Ethics Review Committee (HS-1763). At the enrollment of each GI test, participating subjects were given full details of the study and information on the potential risks. Participants were told to report any adverse event during the test period.

#### Participants

The protocol of ISO 26642:2010 indicated 10 healthy subjects would be selected for a food GI determination, with the assumption of a dropout rate of 20%, at least 12 subjects were required to ensure a data set of 10 subjects at each GI test. In addition, as the GI value of WB (without MLE) was used for determining further relationships of the test groups, 18 participants were enrolled for more solid reference data.

**Fig 1:**
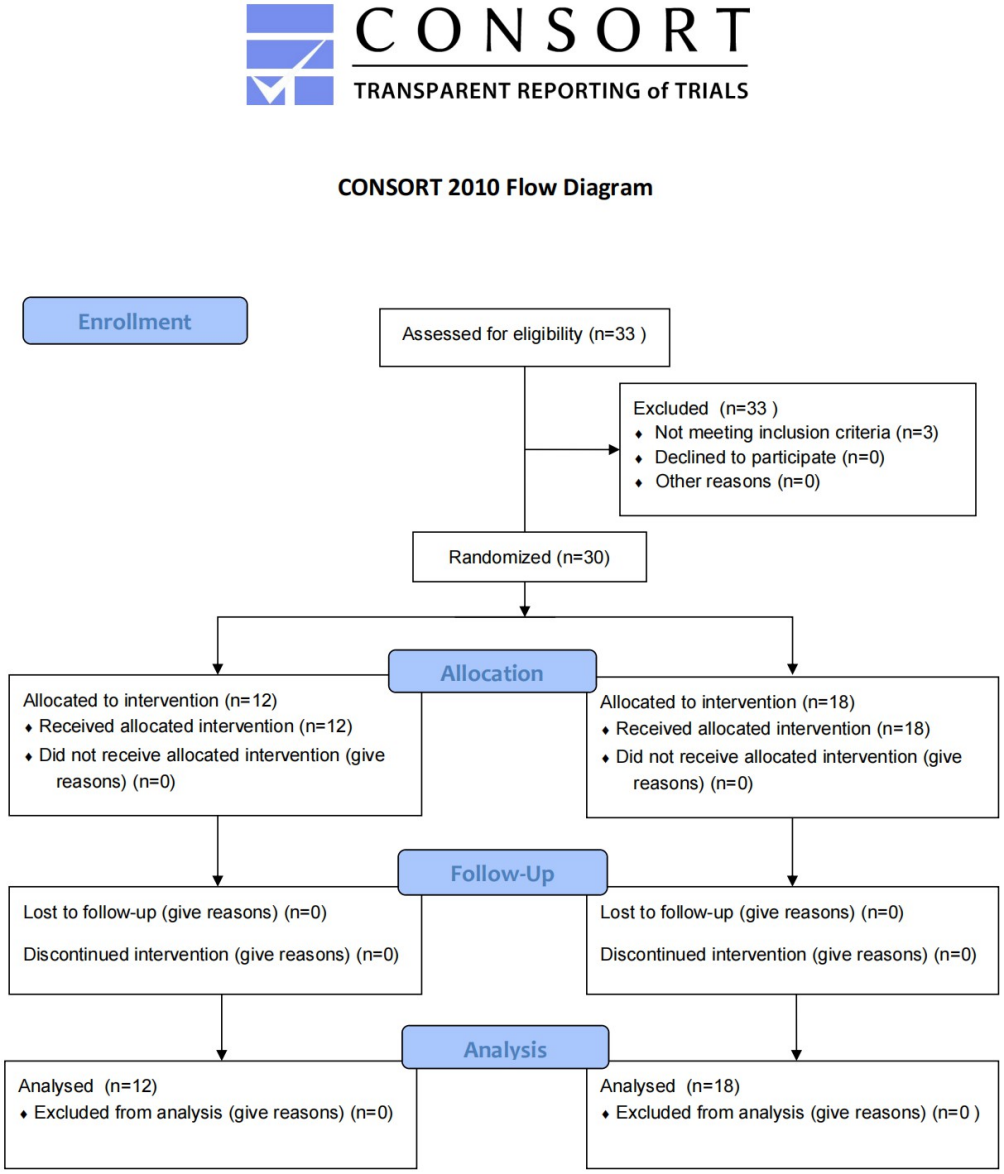
CONSORT flow diagram.

The completed CONSORT flowchart was shown in Fig 1. In total, there were 30 qualified subjects recruited. Wherein, twelve subjects (6 male, 6 female) were randomly selected each time to determine the GI of three MLE-added WBs, 18 participants out of the 30 participants were also randomly selected for the GI determination of WB. Every participant was required to complete a general physical examination and an oral glucose tolerance test (OGTT) that included glucose and insulin examination at the Peking Union Medical College Hospital before being recruited into the study. The inclusion and exclusion criteria complied with the ISO standard requirement.

#### Experimental protocol

The brief study protocol was demonstrated in S2 Fig in S1 File, where each GI test procedure was following the ISO standard.

To evaluate the satiety of WB with different adding levels of MLE, a satiety assessment was conducted for each MLE WB using a method similar to the paper as described by Holts et al. Each participant was asked to interpret self-estimation of satiety and reflected it on a 100 mm visual analog scale (VAS) (S3 Fig in S1 File) at a certain period time from the ingestion of the food. In the current study, each time spot was set to be the same as each of the blood sampling time spots of GI testing.

#### Calculation of GI and satiety assessment values

Samples of blood glucose were collected into heparin anticoagulation tubes from fingertips right before the consumption of the food as fasting blood samples at -5 and 0 min, and at 15, 30, 45, 60, 90, and 120 min after the first bite of the food. Subsequently, the supernatant plasma was separated by centrifugation (MiniSpin, eppendorf^®^) at 5000 rpm for 5 min, and the blood glucose was measured using an automatic biochemistry analyzer (AU480, Beckman Coulter^®^, America). Meanwhile, the satiety assessment was evaluated on a 100 mm visual analog scale (VAS) at each of the same blood sampling time spots. Participants for each food were fixed once after being assigned by random sampling, and 72 h were set as the minimum wash-out period within each consumption occasion.

The average blood glucose response curve was plotted by calculating the mean blood glucose concentrations of all subjects at each time point. The incremental area under the post-prandial blood glucose curve (IAUC), ignoring the area beneath the baseline, was calculated geometrically for each tested food, and the GI was evaluated as a percentage of the mean IAUC of the reference glucose solution consumed by the same subject (GI = IAUC test food/IAUC reference food × 100). When the individual GI values for any subject fell outside the range of values calculated as mean ± 2 SD (standard deviation), this result was considered an outlier and was thus excluded from the mean GI calculation. According to the satiety measurement as described by Holt et al. [21], the marks on the seven-pointed equilateral scale at each time point and the absolute value was measured by a ruler (S3 Fig in S1 File). AUCs of the satiety values of each food or glucose were calculated for statistical analysis.

### Statistical analysis

All statistical analyses were performed using SAS version 9.4 and Origin 2021. Data are presented as mean and SD. Differences in WB characteristics, IAUCs, and GI values were evaluated using one-way analysis of variance (ANOVA) with Tukey’s Multiple Comparison Test at 0.05 level of significance. Comparisons between the postprandial blood glucose concentrations at any time points were made by Student’s t-test, statistical significance was set at *p*<0.05 and *p*<0.01. The least square (LS) method was used to estimate the dose-effect relationship of MLE on reducing the GI value of WB.

## Results

### Effect of MLE on characteristics of WB

#### Chromatic aberration

**Fig 2:**
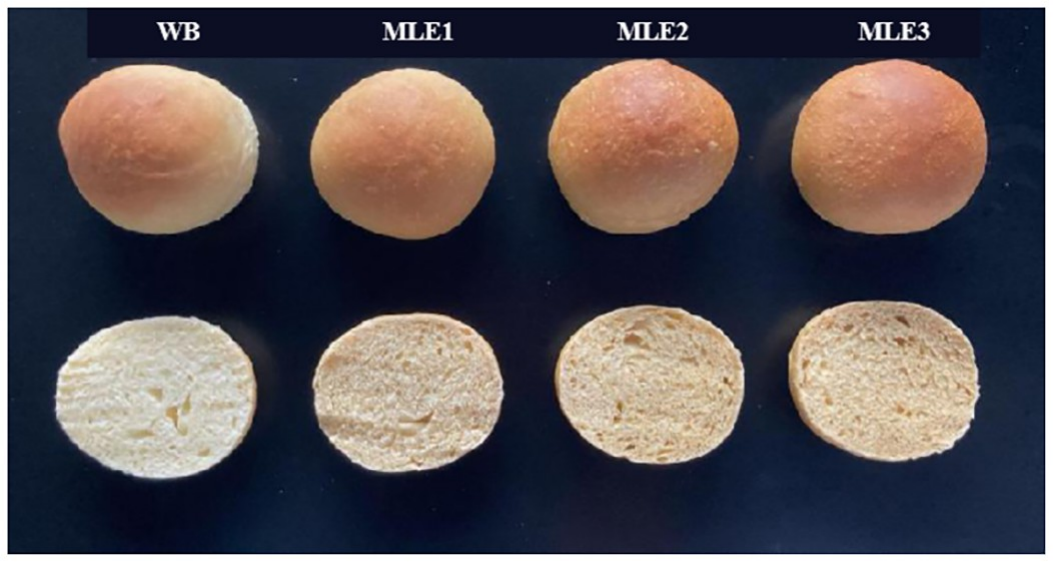
Stomatal structure and appearance of the cross-section of WB with different MLE levels.

As shown in Fig 2, with the increase of MLE content, no obvious changes in the stomatal morphology of WB were found, but the color of the WB core gradually deepened with the increase of MLE amount.

As shown in Tables 1 and 2, compared with WB, the addition of MLE at all three concentrations led to a lower L* value, higher a* value, b* value significantly, and the effects were dose-dependent. This suggests that the addition of MLE decreased the lightness of WB, whereas increased the red and yellow color and the whiteness as well. Similar to the core color, the crust color of MLE WB also showed a lower L*, higher a*, b*, and whiteness compared with WB. Overall, the color of WB became darker with the addition of MLE.

**Table 1:** Color of bread core.

Sample	L*	a*	b*	Whiteness index
WB	82.07±0.07^a^	-2.58±0.07^a^	26.36±0.14^a^	31.99±0.07^a^
MLE1	73.59±0.07^b^	-0.49±00^b^	29.39±0.07^b^	39.52±0.01^b^
MLE2	70.88±00^c^	-0.01±0.07^c^	31.55±0.07^c^	42.93±0.05^c^
MLE3	70.12±0.07^d^	0.33±0.07^d^	31.82±0.07^d^	43.65±0.004^d^

Data in the same column with different superscript letters are significantly different (*p* < 0.05) as assessed by Tukey’s test.

L*, lightness; positive a* represents redness and negative value symbolizes greenness; positive and negative b*signifies yellowness and blueness, respectively.

**Table 2:** Color of bread crust.

Sample	L*	a*	b*	Whiteness index
WB	73.41±0.07^a^	4.34±0.07^a^	28.48±00^a^	39.21±0.04^a^
MLE1	67.73±00^b^	5.26±0.49^b^	31±0.14^b^	45.06±0.16^b^
MLE2	67.96±0.14^c^	4.41±0.07^c^	32.41±0.21^c^	45.78±0.06^c^
MLE3	65.12±0.14^d^	6.33±00^d^	33.12±0.07^d^	48.51±0.05^d^

Data in the same column with different superscript letters are significantly different (*p* < 0.05) as assessed by Tukey’s test.

L*, lightness; positive a* represents redness and negative value symbolizes greenness; positive and negative b*signifies yellowness and blueness, respectively.

#### Textural properties

**Fig 3:**
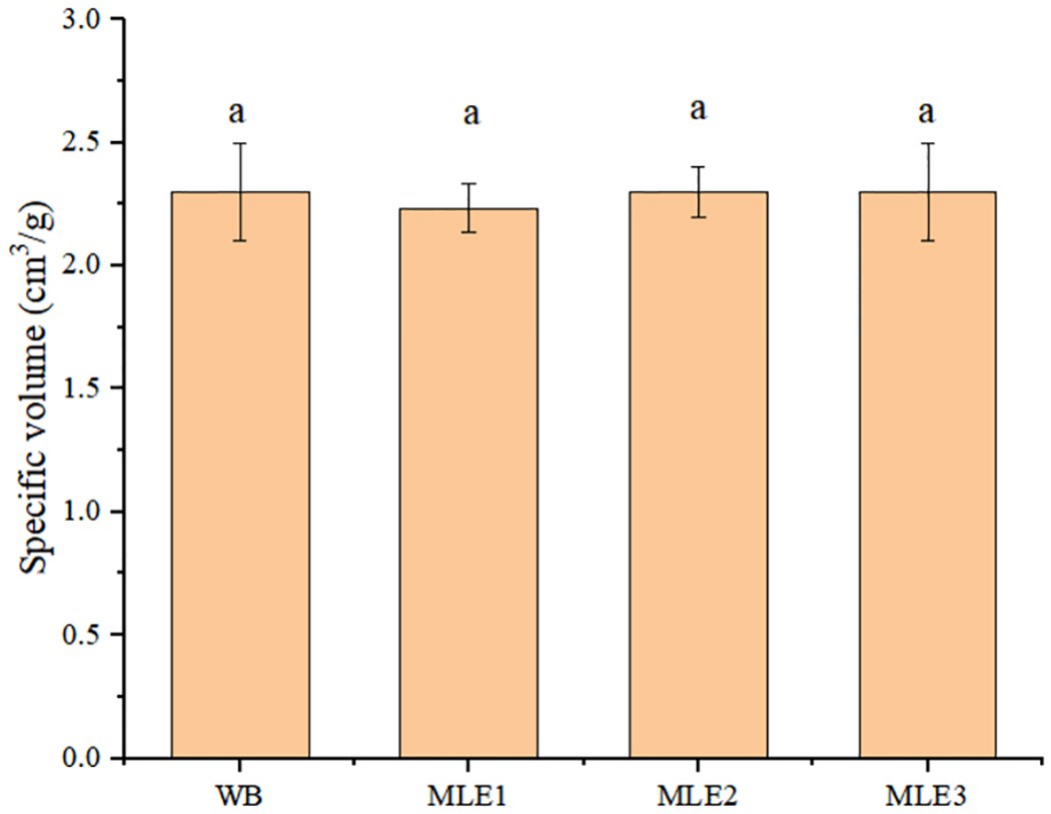
Effect of MLE on the specific volume of WB. Values with the same letter in the same column are not significantly different (*p*>0.05).

The influence of MLE on the specific volume of bread was presented in Fig 3. It indicates that the addition level of MLE at 0.75–1.5g/100g AC did not bring significant change in the specific volume of WB.

Hardness, cohesiveness, and chewiness of WB and MLE WB were obtained from TPA (Table 3). The addition of MLE had little effect on the textural properties of WB, indicating the feasibility of using MLE at the current concentrations for healthy product development without compromising the texture.

**Table 3:** Effect of MLE on textural properties of WB.

Sample	Hardness	Springiness	Cohesiveness	Chewiness	Resilience
WB	1046.54±97.26^a^	0.88±0.05^a^	0.68±0.03^a^	624.49±65.19^a^	0.29±0.02^a^
MLE1	997.31±92.68^a^	0.83±0.05^a^	0.62±0.02^b^	506.15±52.84^a^	0.24±0.01^b^
MLE2	999.82±123.76^a^	0.81±0.06^a^	0.65±0.01^ab^	525.84±60.18^a^	0.26±0.01^a^
MLE3	849.68±64.42^a^	0.84±0.04^a^	0.68±0.03^ac^	480.66±41.74^ab^	0.27±0.02^a^

Data in the same column with different superscript letters are significantly different (*p* < 0.05) as assessed by Tukey’s test.

#### Electronic sensory evaluation

**Fig 4:**
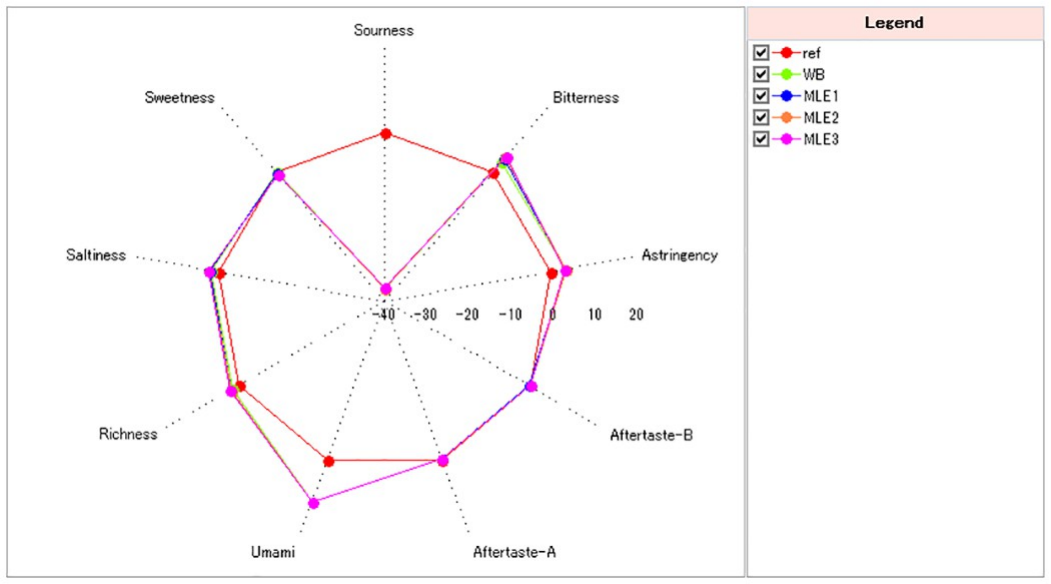
The sensory radar chart of WB with different MLE levels.

E-tongue assessment was performed to evaluate the taste characteristics of bread samples (Table 4). As seen from Fig 4, the values below “- 20” in sourness or “0” in other taste signals were recognized as not being perceived. There were no significant differences (*p*>0.05) of the taste signals in sourness, astringency, and aftertaste-astringency (Table 4). Among other taste dimensions, only the bitterness indicated a light but noticeable difference (more than 1 unit) between WB and MLE WB(MLE1/MLE2/MLE3), while the differences between other variables from each taste were less than “1”. MLE3 showed a positive value for aftertaste-bitterness, however, the signal was quite weak to be perceived. The result indicated that the impact of MLE on changing the flavor of WB was not strong in general. MLE only presented a light bitterness to MLE WB, and brought a very low taste of aftertaste-bitterness, while other tastes didn’t change too much.

**Table 4:** The data of sensory values of WB with different MLE levels.

Test samples	Sourness	Bitterness	Astringency	Aftertaste-B	Aftertaste-A	Umami	Richness	Saltiness
WB	-36.94±0.21^a^	3.11±0.27^a^	3.75±0.1^a^	-0.48±0.04^a^	-0.17±0.03^a^	10.57±0.02^a^	1.66±0.03^a^	1.89±0.01^a^
MLE1	-37.13±0.18^a^	4.29±0.15^b^	3.72±0.08^a^	-0.16±0.03^b^	-0.17±0.01^a^	10.65±0.01^b^	2.01±0.09^b^	2.23±0.01^b^
MLE2	-36.9±0.2^a^	4.72±0.1^c^	3.65±0.06^a^	-0.04±0.05^c^	-0.11±0.02^a^	10.65±0.01^b^	2.17±0.04^c^	2.45±0.02^c^
MLE3	-36.72±0.19^a^	4.91±0.09^c^	3.61±0.07^a^	0.05±0.03^c^	-0.13±0.02^a^	10.64±0.03^b^	2.46±0.11^bc^	2.50±0.01^d^

Data in the same column with different superscript letters are significantly different (*p* < 0.05) as assessed by Tukey’s test.

### *In vitro* results

#### DNJ determination of MLE WB

**Fig 5:**
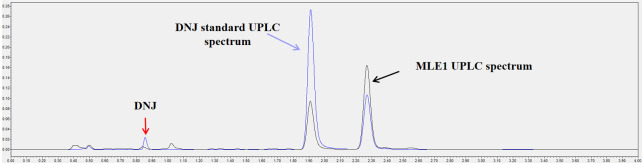
UPLC chromatogram of DNJ standard and MLE1.

The standard solutions were analyzed by UPLC and the results obtained were used to construct a standard curve (R^2^ = 0.9897). The equation from the standard curve was then used to calculate the actual DNJ concentration in the WB and MLE1. The chromatogram from the HPLC analysis was shown in Fig 5. The DNJ retention time was around 1 min. The result of DNJ determination by using HPLC was 7.2±0.4 mg/100 g AC, which compares favorably with the theoretical value (7.5 mg/100 g AC), and showed no significant differences (*p*>0.05).

#### *In vitro* digestion

**Fig 6:**
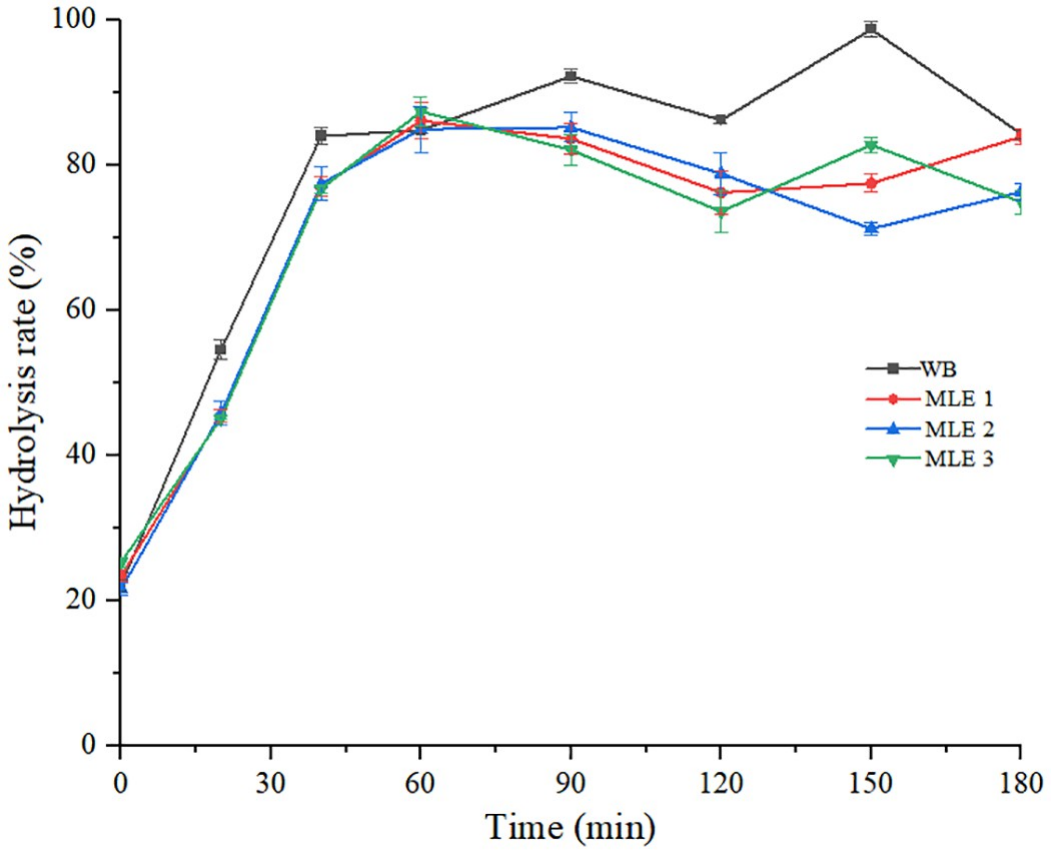
Area under the curve for the *in vitro* starch hydrolysis at 180 min for all types of bread.

**Fig 7:**
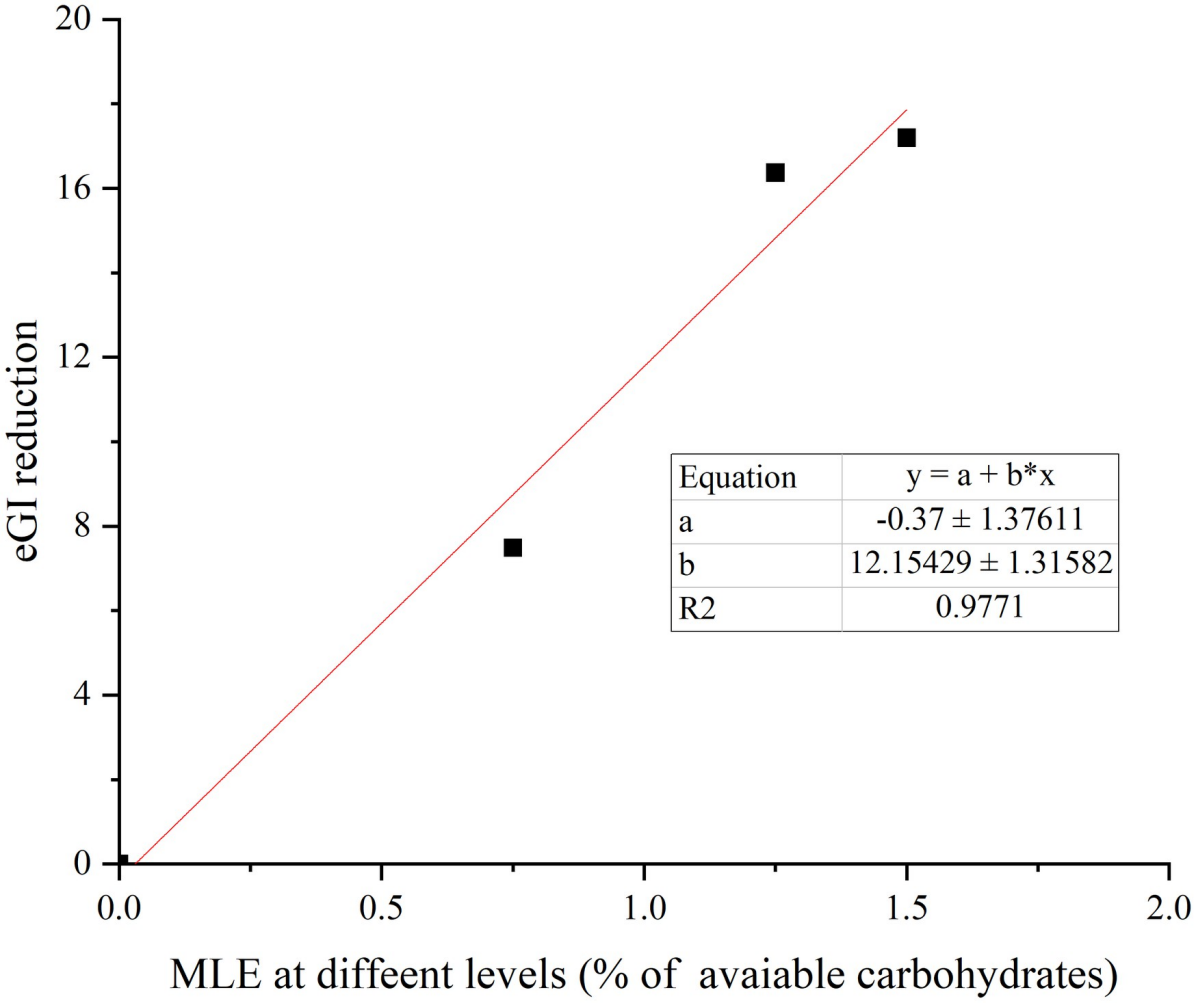
The dose-effect relationship of MLE on reducing eGI value of WB.

The glucose released from WB and MLE WB during 180 min of *in vitro* digestion was shown in Fig 6. The AUCs were significantly lower for MLE WB than for WB. Table 5 showed the calculated HI and the eGI for MLE WB and WB. Adding MLE to WB significantly (*p*<0.05) reduced the HI, and the eGI of WB was also reduced to about 7.9%, 17.3%, and 18.2% by MLE respectively, which showed a clear dose effect-relationship(R^2^ = 0.9771) (Fig 7).

**Table 5:** The HI and eGI of WB and MLE WB.

Test samples	HI	eGI
WB	100.00±2.51^a^	94.40±2.16^a^
MLE1	91.32±1.35^b^	86.91±1.16^b^
MLE2	81.01±1.61^c^	78.03±1.39^c^
MLE3	80.05±1.14^c^	77.20±0.98^c^

Data in the same column with different superscript letters are significantly different (*p* < 0.05) as assessed by Tukey’s test.

### *In vivo* GI

#### Baseline characteristics of the study participants

30 healthy participants were recruited (15 male, 15 female; aged 18 to 37 years old) and all completed the study. The physical characteristics of all participants with complete GI data were presented in S2 Table in S1 File.

#### GI test of WB

**Fig 8:**
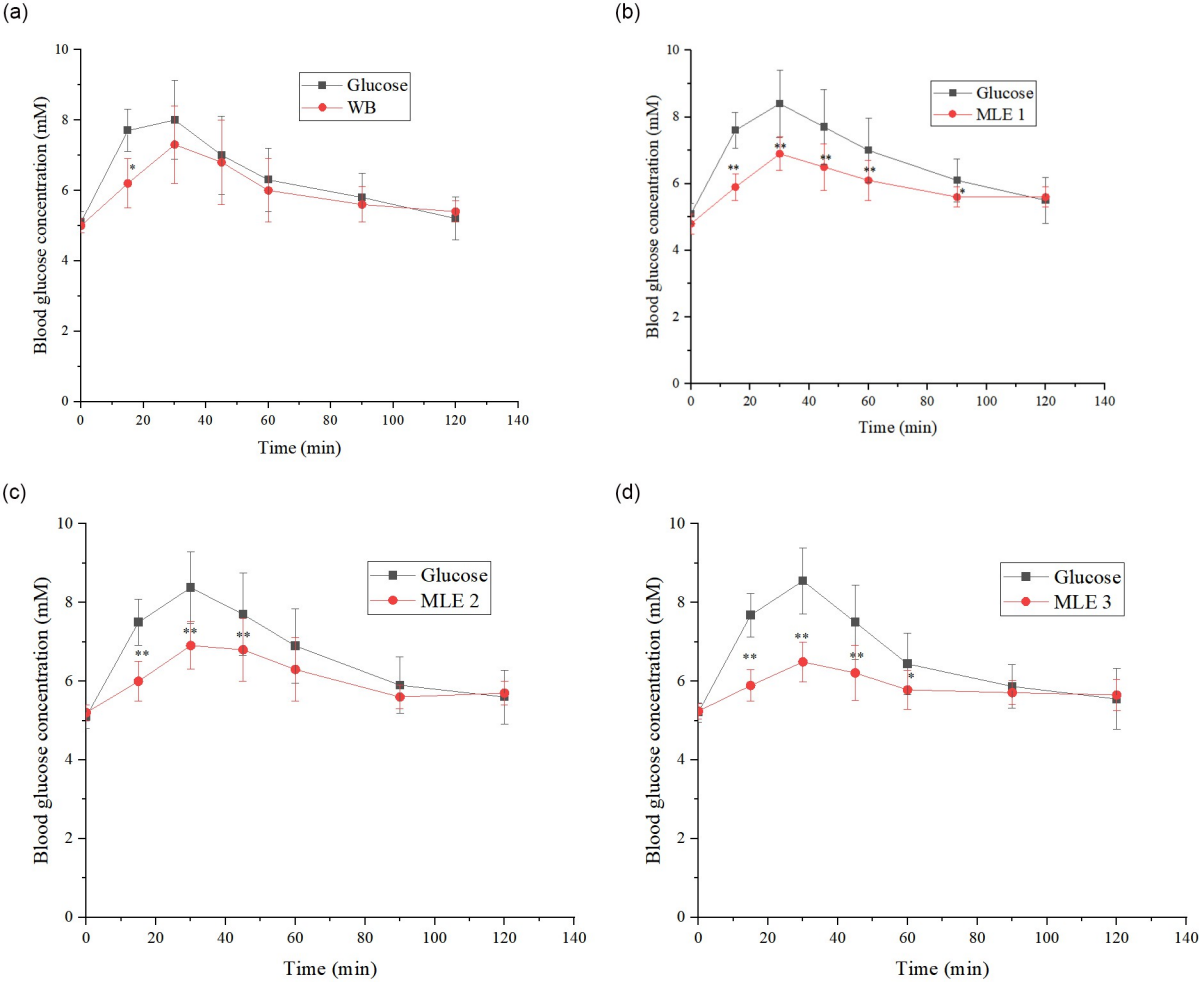
The curves of blood glucose response to glucose, WB and MLE WB. a to d: Glucose levels for WB, MLE1, MLE2, MLE3, and the reference food glucose. Compared to glucose, the asterisk indicates a statistically significant difference (*p<0.05, **p<0.01), as assessed by Student’s t-test.

Eighteen healthy human participants participated in the *in vivo* GI test study of WB. The comparisons of postprandial blood glucose levels between the WB (test food) and glucose (reference food) were first conducted. The blood glucose at each postprandial time point (0, 15, 30, 45, 60, 90, and 120 min) was presented in Fig 8a. After consumption of the test or reference food, the blood glucose level increased progressively to reach a peak at 30 min before gradually falling back to preprandial levels at 120 min. The GI was calculated from the IAUC of WB versus that of glucose. The individual subjects’ GI values of WB were shown to range from 34 to 114, none of the 18 subjects’ data was out of this range (Coefficient of variation≤30%). The mean GI value of WB was finally rounded to 77.

#### GI tests of MLE WB

The GI tests for the experiment groups were conducted independently with 12 randomly selected subjects for each group. The changes in blood glucose relative to the postprandial blood glucose levels within 120 min after the consumption of the test foods were shown in Fig 8b–8d. Compared with glucose alone, WB that contained MLE resulted in less fluctuating postprandial blood glucose responses. There were significant differences (*p*<0.05) in all blood glucose levels between MLE WB and the placebo WB at 15, 30, and 45 min.

**Fig 9:**
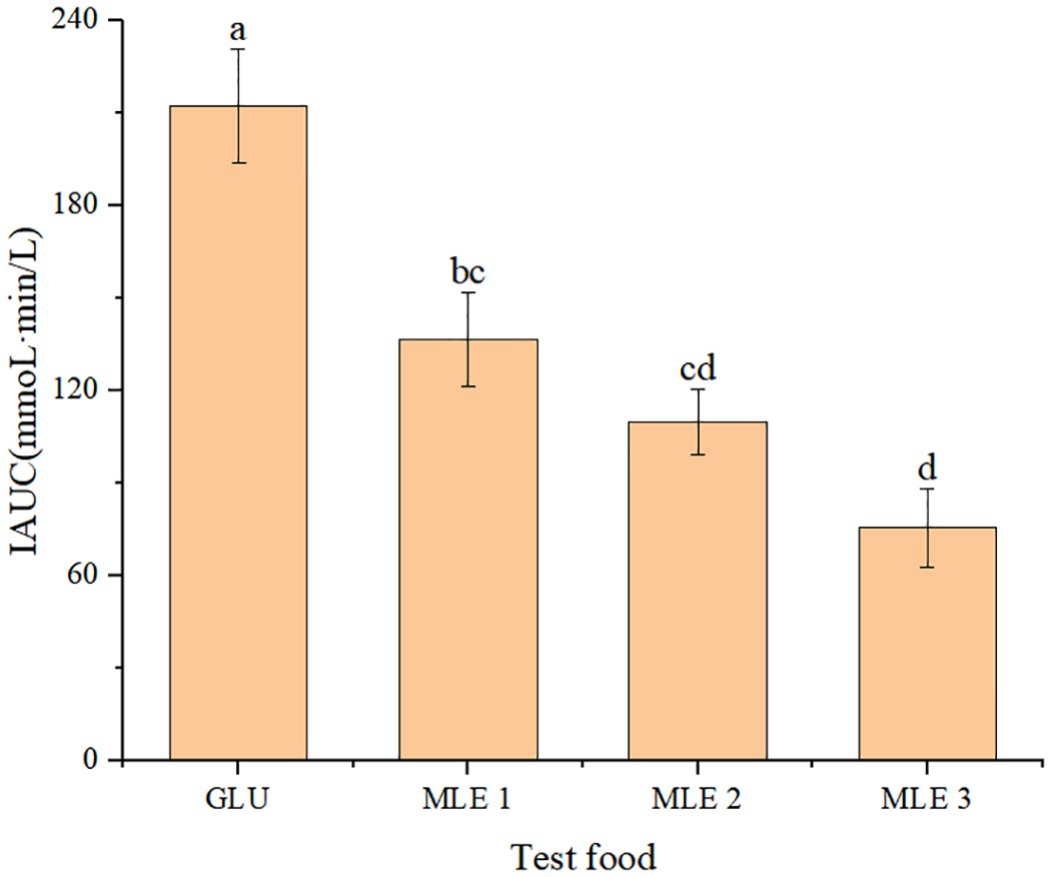
Postprandial blood glucose IAUC for MLE WB and the reference food glucose. Values with the different letter indicates a significant difference (*p*<0.05), as assessed by Tukey’s t-test.

The IAUC over 0–120 min for postprandial blood glucose was obtained (Fig 9). It was clear that the IAUCs of MLE WBs were significantly lower than the reference food glucose, with decrement differences of 36%, 48%, and 64%, respectively (*p*<0.05).

#### Dose-dependent effect of MLE on lowering the GI of WB

**Fig 10:**
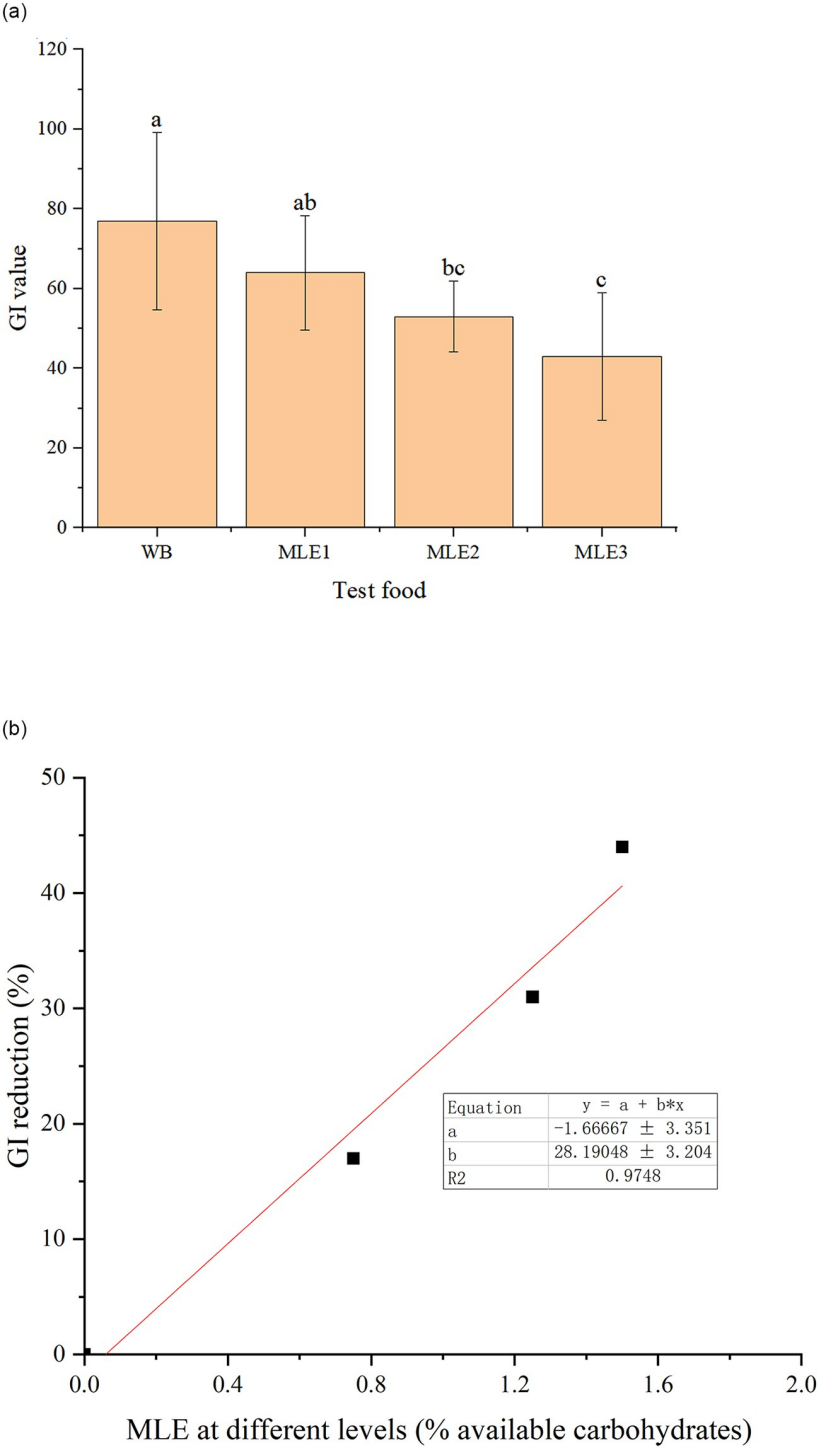
(a) GI value comparison between WB and MLE WB. Values with the different letter indicate a significant difference (*p*<0.05), as assessed by Tukey’s t-test. (b) the dose-effect relationship of MLE on reducing GI value of WB.

Based on the IAUCs results, the calculated GI values for MLE WBs were 64±14 (MLE1), 53±9 (MLE2), and 43±16 (MLE3), respectively. The GI value of WB has been known to be 77, so it could be obtained that the addition of MLE could significantly reduce the GI value of WB by 17% (*p*<0.05), 31% (*p*<0.01), and 44% (*p*<0.01), respectively (Fig 10a). Furthermore, the plot of the dose-depend effect of MLE on reducing GI was obtained (Fig 10b) and the GI reduction effect of MLE on WB was strongly positively correlated with the dose levels (R^2^ = 0.9748), which indicates a good linear fitting relationship between the added level of MLE and the GI value reduction. In addition, the fitting effect was consistent with the *in vitro* eGI results. MLE could inhibit glucose levels with a dose-response relationship *in vitro* and *in vivo*.

### Satiety evaluation

**Fig 11:**
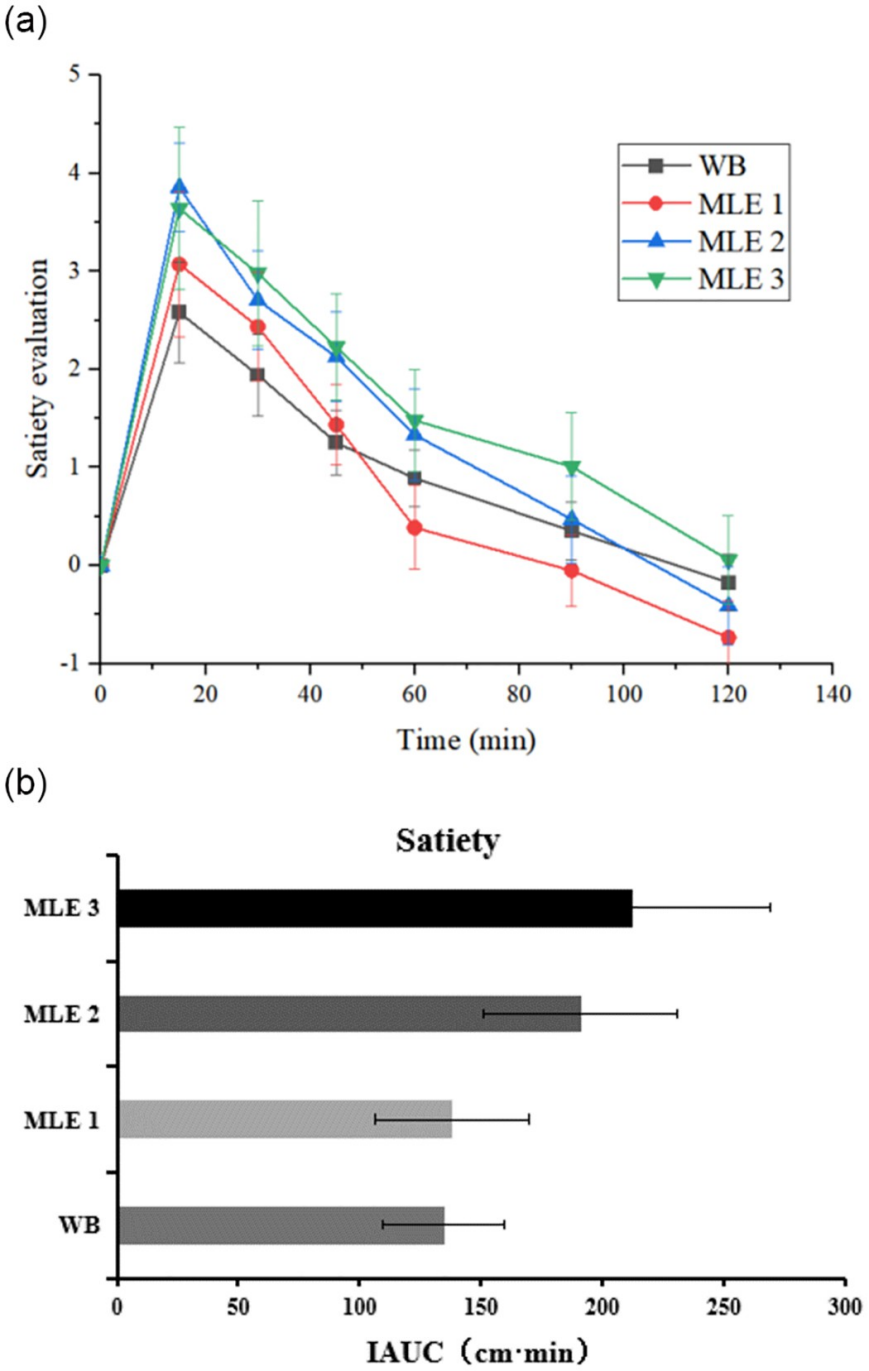
The satiety ratings of WB and MLE WB. (a): Satiety curve; (b): Area under satiety curve.

Based on the VAS data, the relative satiety level was assessed under an isocaloric condition (1285 kJ). The satiety change of each food within 120 min was shown in Fig 11a. All the satiety peaks appeared at 15 min after food intake and gradually decreased as time goes on. In addition, the satiety was higher after eating MLE WB compared with WB (Fig 11b) but did not show any significant difference (*p*>0.05).

Throughout the entire study period, no adverse events or discomfort due to the GI test were reported. Both the glucose and WB (without or with MLE) were well tolerated among every subject accordingly, of all the 30 participants involved in the entire study.

## Discussion

Mulberry leaf has a long history of food and medicinal use in China. Reports show that it is very rich in proteins, amino acids, vitamins, minerals flavonoids, polyphenols, polyose, iminosugars, GABA, etc., and could be used to prevent and cure diabetes and obesity [22]. Previous animal and human studies have demonstrated that MLE could improve glycemic response, and insulin response and reduce the GI of common carbohydrates including maltose, sucrose, maltodextrin, and glucose [10,23]. Previous studies have found that the consumption of biscuits with a beverage of mulberry in the afternoon prevents dinner-induced high increases in blood glucose among young adults [24].

However, most of the current research on MLE is limited to raw food materials and few complex staple food-based studies have been conducted. In addition, few studies have added MLE to products in the processing stage, and then determine the effect of MLE on the postprandial blood glucose response of the product. In this study, WB, a common staple food, was first used as the carrier to explore the reducing effect of MLE on GI. The results demonstrated that MLE could significantly (*p*<0.05) reduce the glycemic response of WB and showed a clear dose-effect relationship. However, we found that the MLE exhibited a lower reducing effect on eGI compared with the *in vivo* results, this may be due to that MLE not only delay the digestion of carbohydrate by inhibiting amylase and α-glucosidase, but also decrease the rise of blood glucose by promoting glucose uptake and utilization [25,26]. The *in vitro* study could only reflect the effect of MLE on inhibiting digestion, while the *in vivo* results represented a more comprehensive effect of MLE. In conclusion, this study demonstrated the activity of MLE in lowering the digestibility and GI of WB, which indicated more practical guidance to the application of MLE. In the present study, MLE was directly added to the other raw materials and underwent fermentation and a high-temperature process. And DNJ, as the main active ingredient in MLE, was not destroyed and still exhibited a good dose-effect relationship *in vivo* study. More interestingly, the addition of MLE had no obvious adverse effect on the textural and other sensory properties of WB, except for bringing a little bitterness. To our knowledge, the current study was the first to explore the possible dose-depend relationship of the glycemic lowering effect of MLE when added to food with complex texture and went through fermentation and baking.

Generally, MLE exhibited the activity of decreasing postprandial glycemia by slowing carbohydrate digestion and glucose absorption [9,10]. The main active ingredient in MLE is DNJ, which belongs to polyhydroxy alkaloids and has been proven to inhibit the absorption of sugar in the small intestine and reduce postprandial blood glucose in some investigations [27]. The absorption and metabolic pathway of the naturally occurring DNJ in plant is different from pure DNJ, and MLE also exhibited a much better effect *in vivo* and was safer than pure DNJ [28]. Moreover, the other active ingredient flavonoids in MLE also might reduce the rate and extent of starch digestion [29,30]. We infer that the reducing effect of MLE on GI was the result of the joint action of multiple active components in MLE. The findings of this study were in good agreement with previous reports that MLE can impede the digestion and absorption of carbohydrates.

A recently published human randomized crossover test has once again proved that DNJ can significantly reduce the glycemic response of some carbohydrates *in vivo* [10]. The results showed that the MLE could reduce the GIs for maltose, sucrose, maltodextrin, and glucose by 53.11%, 33.51%, 31.00%, and 8.12%, respectively [10], at the addition level of 15 mg DNJ/100 g AC. In comparison with our study, the GI of WB could be reduced by 44% when the addition level of MLE was the same ratio. The carbohydrate in WB was mainly consisted of starch and some free sugar produced after starch hydrolysis. Starch was composed of from 6 to 9 D-glucose units linked to one another by an α-1,4-glycosidic bond, and could be firstly hydrolyzed into maltodextrin by ɑ-amylase. And then maltodextrin was mainly broken down into maltose and α-amylodextrin by α-amylase or β-amylase on the mucous membrane of the small intestine. The maltose and α-amylodextrin were subsequently broken down by α-glucosidase or α-dextrinase respectively, ultimately, glucose is generated and absorbed [10]. Since the carbohydrate in white bread mainly includes starch and some free sugars, and the multienzyme, the multistep process for starch may explain why the effect of DNJ on the GI of WB was between maltose and maltodextrin. In additional, another important finding in the present study was the dose-effect correlation (R^2^ = 0.9748) between the addition amount of MLE and GI values. Research showed that MLE that contained 1% DNJ demonstrated a dose-dependent hypoglycemic manner when consumed with maltodextrin at three different levels (R^2^ = 0.9799) [11]. Therefore, the idea that using MLE to design a low GI food by a linear regression formula might be possible.

In the present study, the satiating effect of MLE based on WB also obtained a positive result. Concerning satiety rating, the addition of MLE in WB prolongs the satiety. Although the data did not reach a significant difference, WB with the highest amount of MLE increased the late postprandial satiety by 50%, compared with the reference WB. None of the former articles on meal supplementation with MLE have evaluated satiety, and the current finding added new knowledge both to the specific effect of MLE on satiety and to the more general relation between GI and satiety. Food with a lower GI is usually digested more slowly and could provide higher satiety. It has been studied that increasing the content of resistant starch by adding procyanidins in food may be related to slower digesting, delayed gastric emptying, and prolonged satiety [31]. Therefore, the digestion enzyme inhibition of MLE might produce undigested resistant carbohydrates, which could enter the ileum, triggering the "ileal braking" mechanism to transmit the satiety signal to the brain, thus reducing appetite and food intake [32]. The above may be the reason why MLE WB could provide higher satiety than WB and showed a dose-effect trend. However, more underlying in-depth mechanisms still need to be explored in the future.

## Conclusions

We have demonstrated that adding MLE (Sangduoan^®^) in WB could significantly reduce its GI value, and there was an obvious dose-depend relationship between the amount of MLE and GI values (R^2^ = 0.9748), which was also supported by *in vitro* results. In addition, MLE did not bring any adverse effect on the textural and sensory properties of WB except bringing a little bitterness. The findings of the present study illustrated the possibility that supplementation of MLE into real food could be practical and would potentially help to suppress postprandial blood glucose levels. Future studies might need to explore the effectiveness of MLE when applied to different foods which contain more complex carbohydrates.

## Supporting information

S1 Checklist CONSORT 2010 checklist of information to include when reporting a randomised trial*. (DOC)

S1 File Supplementary figures and tables. (DOC)

S2 File (PDF)

S1 Protocol Trial protocol. (PDF)

## References

[1] Subramanian SS.Stability ball on glycemic control in type 2 diabetes mellitus. IOSR J. Appl. Chem. 2012;1(3):10–13. doi: 10.9790/5736-0131013

[2] Wolever TM, Jenkins DJ, Jenkins AL, Josse RG. The glycemic index: methodology and clinical implications. Am J Clin Nutr. 1991;54(5):846–54. doi: 10.1093/ajcn/54.5.846 1951155

[3] Smith RN, Braue A, Varigos GA, Mann NJ. The effect of a low glycemic load diet on acne vulgaris and the fatty acid composition of skin surface triglycerides. J Dermatol Sci. 2008;50(1):41–52. doi: 10.1016/j.jdermsci.2007.11.005 18178063

[4] Stevenson E, Williams C, McComb G, Oram C. Improved recovery from prolonged exercise following the consumption of low glycemic index carbohydrate meals. Int J Sport Nutr Exe. 2005;15(4):333–49. doi: 10.1123/ijsnem.15.4.333 16286667

[5] Yin Z, Zhang W, Feng F, Zhang Y, Kang W. Food Sci. Hum. Wellness. 2014, 3, 136.

[6] Lochocka K, Bajerska J, Glapa A, Fidler-Witon E, Nowak JK, Szczapa T, et al. Green tea extract decreases starch digestion and absorption from a test meal in humans: a randomized, placebo-controlled crossover study. Sci Rep-Uk. 2015;5:12015. doi: 10.1038/srep12015 26226166 PMC4520190

[7] Goto T, Horita M, Nagai H, Nagatomo A, Nishida N, Matsuura Y, et al. Tiliroside, a glycosidic flavonoid, inhibits carbohydrate digestion and glucose absorption in the gastrointestinal tract. Mol Nutr Food Res. 2012;56(3):435–45. doi: 10.1002/mnfr.201100458 22173993

[8] Hua F, Zhou P, Wu HY, Chu GX, Xie ZW, Bao GH. Inhibition of alpha-glucosidase and alpha-amylase by flavonoid glycosides from Lu’an GuaPian tea: molecular docking and interaction mechanism. Food Funct. 2018;9(8):4173–83. doi: 10.1039/c8fo00562a 29989631

[9] Chukwuma CI, Mopuri R, Nagiah S, Chuturgoon AA, Islam MS. Erythritol reduces small intestinal glucose absorption, increases muscle glucose uptake, improves glucose metabolic enzymes activities and increases expression of Glut-4 and IRS-1 in type 2 diabetic rats. Eur J Nutr. 2018;57(7):2431–44. doi: 10.1007/s00394-017-1516-x 28770335

[10] Wang R, Li Y, Mu W, Li Z, Sun J, Wang B, et al. Mulberry leaf extract reduces the glycemic indexes of four common dietary carbohydrates. Medicine. 2018;97(34):e11996. doi: 10.1097/MD.0000000000011996 30142838 PMC6113008

[11] Lown M, Fuller R, Lightowler H, Fraser A, Gallagher A, Stuart B, et al. Mulberry-extract improves glucose tolerance and decreases insulin concentrations in normoglycemic adults: Results of a randomised double-blind placebo-controlled study. Plos One. 2017;12(2):e172239. doi: 10.1371/journal.pone.0172239 28225835 PMC5321430

[12] Wang T, Li C, Zhang H, Li J. Response Surface Optimized Extraction of 1-Deoxynojirimycin from Mulberry Leaves (*Morus alba* L.) and Preparative Separation with Resins. Mol. 2014; 19(6): 7040–7056. doi: 10.3390/molecules19067040 24886934 PMC6271188

[13] Zhu F, Sakulnak R, Wang S. Effect of black tea on antioxidant, textural, and sensory properties of Chinese steamed bread. Food Chem. 2016;194:1217–23. doi: 10.1016/j.foodchem.2015.08.110 26471674

[14] Ding S, Peng B, Li Y, Yang J. Evaluation of specific volume, texture, thermal features, water mobility, and inhibitory effect of staling in wheat bread affected by maltitol. Food Chem. 2019;283:123–30. doi: 10.1016/j.foodchem.2019.01.045 30722851

[15] Caine WR, Aalhus JL, Best DR, Dugan ME, Jeremiah LE. Relationship of texture profile analysis and Warner-Bratzler shear force with sensory characteristics of beef rib steaks. Meat Sci. 2003;64(4):333–9. doi: 10.1016/S0309-1740(02)00110-9 22063112

[16] Zhi R, Zhao L, Zhang D. A Framework for the Multi-Level Fusion of Electronic Nose and Electronic Tongue for Tea Quality Assessment. Sensors (Basel). 2017; 17(5): 1007. doi: 10.3390/s17051007 28467364 PMC5469530

[17] Wulandari YRE, Prasasty VD, Rio A, Geniola C. Determination of 1-deoxynojirimycin content and phytochemical profiles from young and mature mulberry leaves of morus spp. OnLine J. Biol. Sci. 2019;19: 124–131. https://thescipub.com/abstract/10.3844/ojbsci.2019.124.131.

[18] Brodkorb A, Egger L, Alminger M, Alvito P, Assuncao R, Ballance S, et al. INFOGEST static *in vitro* simulation of gastrointestinal food digestion. Nat Protoc. 2019;14(4):991–1014. doi: 10.1038/s41596-018-0119-1 30886367

[19] Ding Q, Nie S, Hu J, Zong X, Li Q, Xie M. *In vitro* and *in vivo* gastrointestinal digestion and fermentation of the polysaccharide from Ganoderma atrum. Food Hydrocolloids. 2017;63: 646–645. doi: 10.1016/j.foodhyd.2016.10.018

[20] Granfeldt Y, Bjrck I, Drews A, Tovar J. An *in vitro* procedure based on chewing to predict metabolic response to starch in cereal and legume products. Eur. J. Clin. Nutr. 1992;46(3):649–660. 1396482

[21] Holt SH, Miller JC, Petocz P, Farmakalidis E. A satiety index of common foods. Eur. J. Clin. Nutr. 1995;49(9): 675–690. 7498104

[22] Jozefczuk J, Malikowska K, Glapa A, Stawinska-Witoszynska B, Nowak JK, Bajerska J, et al. Mulberry leaf extract decreases digestion and absorption of starch in healthy subjects-A randomized, placebo-controlled, crossover study. Adv Med Sci-Poland. 2017;62(2):302–6. doi: 10.1016/j.advms.2017.03.002 28501729

[23] Thondre PS, Lightowler H, Ahlstrom L, Gallagher A. Mulberry leaf extract improves glycemic response and insulaemic response to sucrose in healthy subjects: results of a randomized, double blind, placebo-controlled study. Nutr Metab. 2021;18(1):41. doi: 10.1186/s12986-021-00571-2 33858439 PMC8047566

[24] Kim JY, Ok HM, Kim J, Park SW, Kwon SW, Kwon O. Mulberry leaf extract improves postprandial glucose response in prediabetic subjects: a randomized, double-blind placebo-controlled trial. J Med Food. 2015;18(3):306–13. doi: 10.1089/jmf.2014.3160 25343729

[25] Bae UJ, Jung ES, Jung SJ, Chae SW, Park BH. Mulberry leaf extract displ21ays antidiabetic activity in *db/db* mice *via* Akt and AMP-activated protein kinase phosphorylation. Food Nutr. Res. 2018; 62:1473. doi: 10.29219/fnr.v62.1473 30150922 PMC6109265

[26] Liu Y, Li X, Xie C, Luo X, Bao Y, Wu B, et al. Prevention Effects and Possible Molecular Mechanism of Mulberry Leaf Extract and its Formulation on Rats with Insulin-Insensitivity. Plos One. 2016; 11(4): e152728. doi: 10.1371/journal.pone.0152728 27054886 PMC4824359

[27] Kwon HJ, Chung JY, Kim JY, Kwon O. Comparison of 1-deoxynojirimycin and aqueous mulberry leaf extract with emphasis on postprandial hypoglycemic effects: *in vivo* and *in vitro* studies. J Agric Food Chem. 2011; 59(7): 3014–3019. doi: 10.1021/jf103463f 21370820

[28] Nakagawa K, Kubota H, Kimura T, Yamashita S, Tsuzuki T, Oikawa S, et al. Occurrence of orally administered mulberry 1-deoxynojirimycin in rat plasma. J Agr Food Chem. 2007;55(22):8928–8933. doi: 10.1021/jf071559m 17914870

[29] Kan L, Capuano E, Fogliano V, Oliviero T, Verkerk R. Tea polyphenols as a strategy to control starch digestion in bread: the effects of polyphenol type and gluten. Food Funct. 2020;11(7):5933–5943. doi: 10.1039/d0fo01145b 32567616

[30] Kan L, Oliviero T, Verkerk R, Fogliano V, Capuano E. Interaction of bread and berry polyphenols affects starch digestibility and polyphenols bio-accessibility. Funct. Foods. 2020; 68:103924. doi: 10.1016/j.jff.2020.103924

[31] Liu R, Xu C, Cong X, Wu T, Song Y, Zhang M. Effects of oligomeric procyanidins on the retrogradation properties of maize starch with different amylose/amylopectin ratios. Food Chem. 2017;221:2010–2017. doi: 10.1016/j.foodchem.2016.10.131 27979193

[32] Mela DJ, Cao XZ, Dobriyal R, Fowler MI, Lin L, Joshi M, et al. The effect of 8 plant extracts and combinations on post-prandial blood glucose and insulin responses in healthy adults: a randomized controlled trial. Nutr Metab. 2020;17:51. doi: 10.1186/s12986-020-00471-x 32647531 PMC7336677

