# Supplementary material for: The impact of mulberry leaf extract at three different levels on reducing the glycemic index of white bread
Source: PLoS One. 2023 Aug 10;18(8):e0288911. doi: 10.1371/journal.pone.0288911 (PMC10414662; doi:10.1371/journal.pone.0288911)
Supplement: S1 File — (DOC) [file pone.0288911.s002.doc]

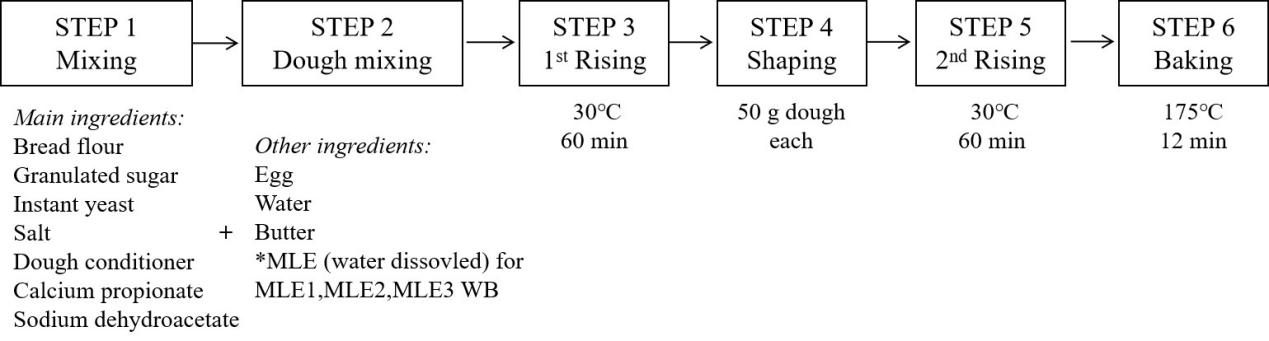


**S1 Fig.** Flowchart of test food preparation


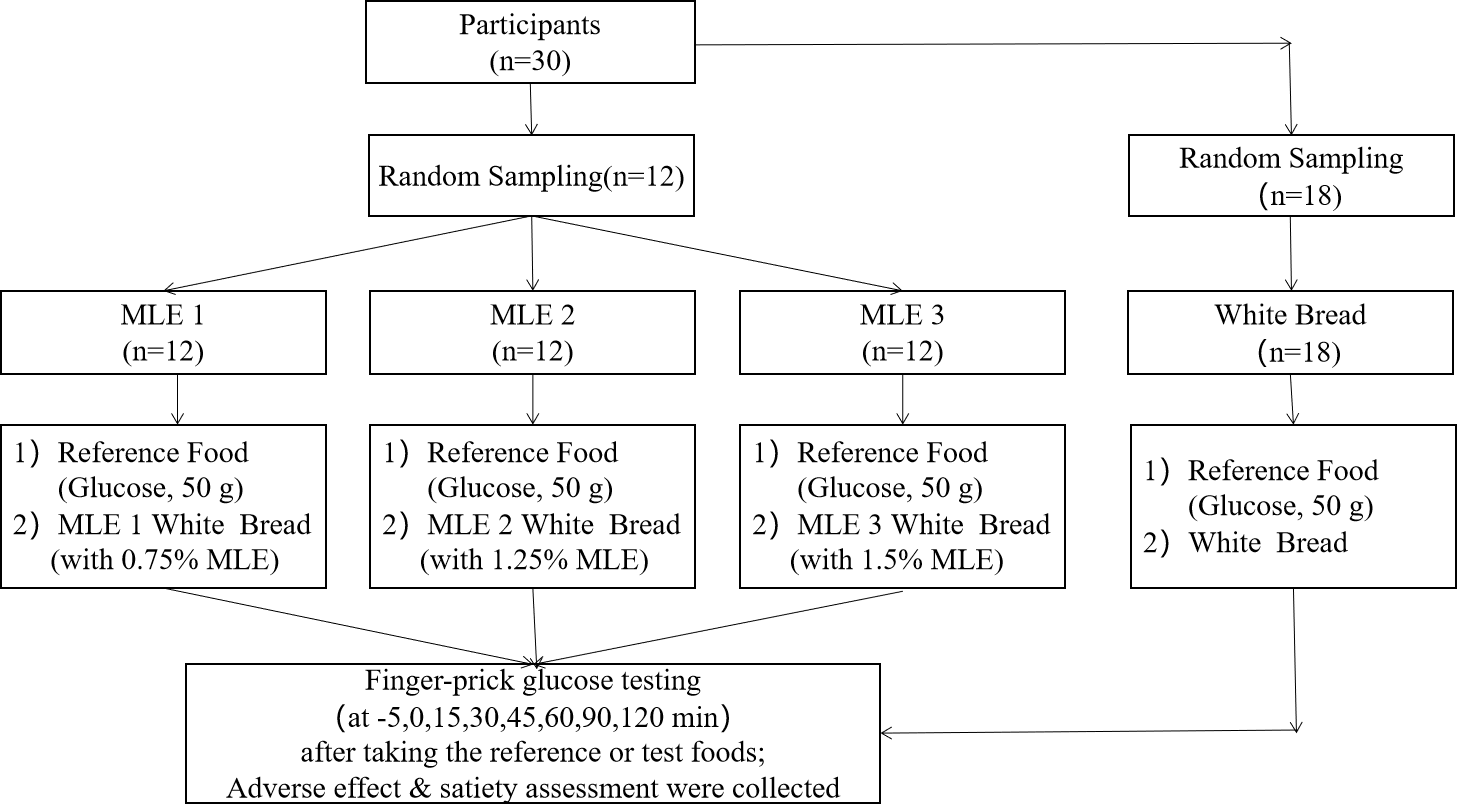


**S2 Fig.** Study protocol of the four experiment groups


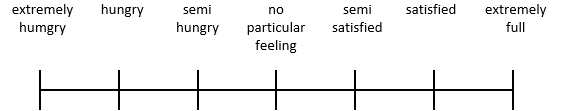


**S3 Fig.** The rating scale used to assess subjective satiety

**S1 Table** Compositions of simulated digestive juices in 500 mL deionized water

| Oral fluid | Gastric juice | Intestinal juice |
| --- | --- | --- |
| 563.23 mg KCl  251.6 mg KH_2_PO_4_  0.571 g NaHCO_3_  15.25 mg MgCl_2_(H_2_O)_6_  2.88 mg (NH_4_)_2_CO_3_  0.110 g CaCl_2_(H_2_O)_2_ | 1.55 g NaCl  0.55 g KCl  0.075 g CaCl_2_  0.3 g NaHCO_3_  5 mL CH_3_COONa  (1 mol/L，pH 5.0) | 0.675 g NaCl  81.25 mg KCl  41.25 mg CaCl_2_·2H_2_O |
| Adjuncts | | |
| 7.50 g α-amylase | 118.0 mg pepsin | 10 g Bile salts  8.75 g pancreatin |
| pH | | |
| 7.0 ± 0.2 | 2.0 ± 0.2 | 7.0 ± 0.2 |

**S2 Table** Physical characteristics of study population (n=23)

| **Variable** | **Mean±SD** |
| --- | --- |
| Age (year) | 27.9±5.7 |
| Height (m) | 1.7±0.1 |
| Weight (kg) | 61.4±11.0 |
| BMI (kg/m^2^) | 21.5±1.7 |
| Waist circumference (cm) | 78.4±7.4 |
