# Supplementary material for: The impact of mulberry leaf extract at three different levels on reducing the glycemic index of white bread
Source: PLoS One. 2023 Aug 10;18(8):e0288911. doi: 10.1371/journal.pone.0288911 (PMC10414662; doi:10.1371/journal.pone.0288911)
Supplement: S2 File — (PDF) [file pone.0288911.s003.pdf]

临床试验研究计划书

中国临床试验注册中心申请用

1. 诚信申明

本研究保证操作严格按照试验规程和数据记录的真实性; 本研究不存在任何利益冲突。

2. 研究题目

添加了三个不同剂量的食品原料桑叶提取物（桑多安）的白面包的血糖生成指数研究

3. 研究背景

常见食物中含有碳水化合物非常普遍，且种类众多， 根据是否在体内利用可分为，可利用和不可利用碳水化合物两类，其不同的组成模式与血糖生成指数（GI）有直接的关联。GI 值是衡量食物引起人体餐后血糖反应的有效指标，是基于人体数据的食物生理学参数。经过 40 年的研究发现，低（GI＜55）的食物对于慢病（糖尿病、高血脂、肥胖等）营养干预有十分显著的效果，对于运动营养、皮肤营养、儿童认知发育等方面也有积极作用。因此探究不同类型膳食纤维、糖醇等碳水化合物成分与可利用碳水化合物的组合模式，与食品食入后可能引起的血糖反应的关系，具有重要的临床意义。推动低 GI 膳食有利于食品营养学属性研究的发展，间接促进消费者开展消费选择和自我饮食指导。

目前国际已经发布 GI 测定的 IS 标准，ISO26642:2010。鉴于慢病的流行现状及糖尿病防控、治疗中膳食干预的重要作用，以及众多产品企业的研究需求，本研究的目的是采用国际标准试验方法 ISO26642:2010 及标准流程，系统评估不同组 成模式碳水化合物产品/食品的 GI 值及其对人体短期血糖代谢的影响。

近年来，针对血糖生成指数食物和低 GI 产品的研发日益增多，其中，针对功能性的植物提取物类成分对糖代谢途径的作用以及在低 GI 食品研发中是被认为很有潜力和研究意义的领域。其中的桑叶提取物，具有有效成分 1-脱氧野尻霉素（DNJ）。它属于一种天然的生物碱偶氮糖，在糖代谢中可视为一种竞争性的α-葡萄糖苷酶抑制剂——竞争性地抑制碳水化合物底物与α-葡萄糖苷酶的结合，减缓小肠中摄入的碳水化合物的分解和吸收，从而抑制餐后血糖的激烈波动。

目前，国内外对桑叶提取物的功效研究并不多，在真实食物中添加不同浓度的桑叶提取物的 GI 相关研究仍较为空白，因此，本实验属于探索性研究桑叶提取物以面包为食物媒介对血糖生成指数的影响效果。

4. 研究目的：

探究桑叶提取物的添加对于白面包血糖生成指数的影响，以及不同浓度的桑叶提取物添加量对于面包血糖生成指数影响作用的程度。此外，探索饱腹感相关评价的变化情况。

5. 纳入和排除标准

纳入标准：

（1）年龄在 18-40 岁健康成年人，男女各半；

（2）体重正常，BMI 在 18.5-24.0 kg/m2 之内，且无代谢性疾病、消化性疾病和内分泌性疾病；

（3）无糖尿病史，未使用降血糖药物；

（4）无食物过敏史和不耐受史，无晕血史；

（5）近 3 个月内无服用影响糖耐量的药物。口服稳定剂量避孕药、乙酰水杨酸、甲状腺素、维生素和矿物质补充剂或药物治疗高血压或骨质疏松症的药物是可以接受的；

（6）能够忍受至少 10 h 的禁食。

排除标准：

（1）已知糖尿病史或使用抗高血糖药物或胰岛素治疗糖尿病及相关病症;

（2）在过去 3 个月内有需要住院的重大医疗或手术事件;

（3）存在影响营养物质消化和吸收的疾病或药物;

（4）使用类固醇、蛋白酶抑制剂或抗精神病药（所有这些药物均对葡萄糖代谢和体脂分布有重要影响）。

6. 设计方案/模式图

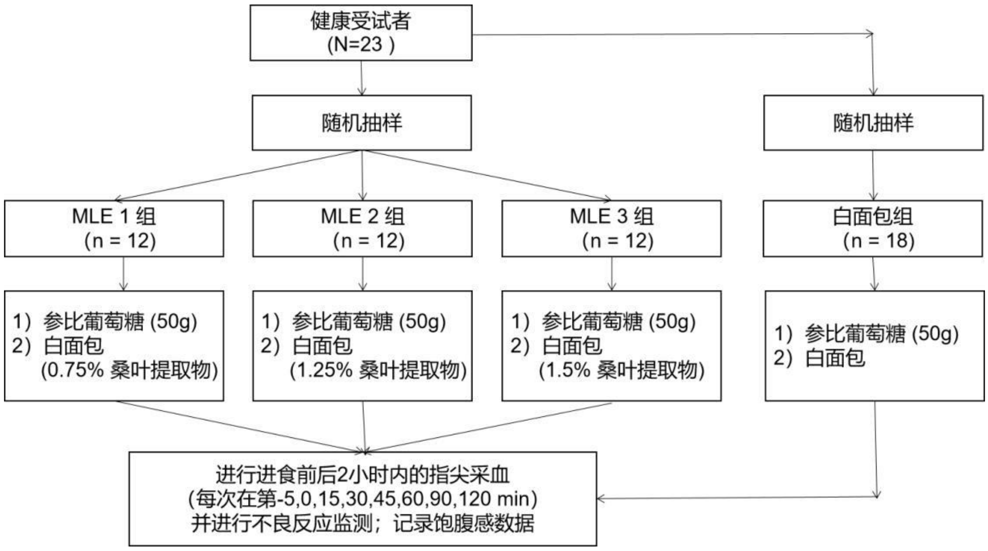

本研究目的类型为探索性研究。具体研究设计见上图示。

7. 样本量估算

根据血糖生成指数 ISO 26642 执行标准，10 人为单次血糖生成指数最低人数。由样本量及脱落率，以及志愿者来源的实际情况。每组实验设计为至少 12 名受试者参与血糖生成指数测试。

8. 测量指标

测量指标为血糖系列指标，以及由餐后血糖变化测算的血糖生成指数。

9. 伦理考量

该研究通过中国医学科学院北京协和医院伦理审查委员会的审批，伦理审查文号：HS-1763（见附件）。

10. 标准操作流程

根据受试者血糖生成指数 ISO 26642 执行标准执行。

本实验遵照国际统一标准，采用一次性采血针指尖取血。

【每个 GI 测试周期】

测试前三日受试者规律作息，正常饮食；测试前一日晚餐避免高膳食纤维及高糖食物，不可饮酒，22:00 前开始禁食；测定当日清晨避免剧烈运动。志愿者抵达测试现场后静坐 10 min 后开始试食测定。间隔 5 min 采集 2 次空腹血样（-5 min 及 0 min），采集结束开始进食，在 5~10 min 内进食完全部受试物及一杯 250 mL 的水，从第一口进食时间开始计时。分别于餐后 15、30、45、60、90 和 120 min 采集血样。

测定周期包括 3~4 次独立试食测定，其中参考食物 2~3 次，受试食物 1 次，采用随机设计。每次独立试食测定间隔≥72 h，受试食物安排在 2 次参考食物测定之间进行。

此外，每次进行 GI 测试时，受试者对在采集血样时的饱腹感进行视觉量表评价。

11. 统计分析方法

以葡萄糖标准物或其他拟定参考实物为对照时，通过比较相同时间点测试食物引起的相应血糖水平变化，进行统计学分析。

数据采用 Excel 绘制图表，并进行统计学分析，差异显著性分析采用 T 检验，$P<0.05$ 为存在显著差异性，$P<0.01$ 为存在极显著差异性。

- 血糖测试方法

（1）血糖测试设备：贝克曼 AU480 全自动生化仪（CV<3.6%）。

（2）采血方式：指尖采血收集血样。

- GI 计算方法

以时间为横坐标，各时点血糖值为纵坐标，制作血糖应答曲线，计算血糖曲线下面积，并以葡萄糖参照物的 GI 值为 100（白面包的 GI 值为 71），计算测试食物 GI 值 I_{mean}。

$$I_{mean}=\text{受试食物 IAUC/葡萄糖参照物 IAUC}\times 100$$

- 饱腹感评价方法

饱腹感比对采用 VAS 量表，对受试者在进食前以及进食后 2 小时内 8 个时间点（0 min、15 min、30 min、45 min、60min、90 min 和 120 min）进行主观感受评价进行数据收集及处理。

12. 发表计划

本研究计划在 2022 年进行文献投稿及发布
