# Supplementary material for: The impact of mulberry leaf extract at three different levels on reducing the glycemic index of white bread
Source: PLoS One. 2023 Aug 10;18(8):e0288911. doi: 10.1371/journal.pone.0288911 (PMC10414662; doi:10.1371/journal.pone.0288911)
Supplement: S1 Protocol — (PDF) [file pone.0288911.s004.pdf]

Clinical trial study protocol

For ChiCTR application

1. Declaration of good faith

This study ensures that the operation is in strict accordance with the test procedures and the authenticity of data records, and there is no conflict of interest in this study.

2. Research topic

The impact of mulberry leaf extract at three different levels on reducing the glycemic index of white bread.

3. Research background

Carbohydrates are very commonly found in many kinds of food. According to whether they can be utilized by the body, they are divided into two types: available and unavailable carbohydrates. Their different composition patterns are directly related to the glycemic index (GI). GI is an effective index to measure the post-prandial blood glucose response caused by food, and is a food physiological parameter based on human data. After 40 years of research, it is found that low (GI<55) food has a very significant effect on nutrition intervention for chronic diseases (diabetes, hyperlipidemia, obesity, etc.), and also has a positive effect on sports nutrition, skin health, children's cognitive development, etc. Therefore, it is of great clinical significance to explore the combination pattern of different types of dietary fiber, sugar alcohol and other carbohydrate components with available carbohydrates, and the relationship between the combination pattern and the possible blood glucose response after food ingestion. The promotion of low GI diet is conducive to the development of food nutrition attribute research, and therefore to promote consumers to make consumption choice.

At present, the ISO26642:2010 is the international method for food GI determination. In view of the epidemic status of chronic diseases, the important role of dietary intervention in the prevention, control and treatment of diabetes, and the research needs of many product enterprises, the purpose of this study is to systematically evaluate the GI value of different composition modes of carbohydrate products/foods and their impact on short-term blood glucose metabolism of human body by following the international standard test method ISO26642:2010 and standard process.

In recent years, the research and development of low GI products have increased day by day. Among them, the role of functional plant extracts on glucose metabolism pathway and the research and development of low GI foods are considered to have great potential and research significance. The mulberry leaf extract has the effective component 1-deoxynojirimycin (DNJ). It belongs to a natural alkaloid azo sugar, which can be regarded as a competitive α-Glucosidase inhibitor — Competitive inhibition of carbohydrate substrate and α-binding of glucosidase, therefore to slow down the decomposition and absorption of carbohydrates in the small intestine, thus inhibiting the drastic fluctuation of postprandial blood sugar.

At present, there are not many studies on the efficacy of mulberry leaf extract at home and abroad, and the GI related research of adding different concentrations of mulberry leaf extract to real food is still relatively blank. Therefore, this experiment is an exploratory study of the effect of mulberry leaf extract using bread as food medium on blood glucose production index.

4. Research purpose:

This study aims to explore the addition of mulberry leaf extract on the glycemic index and sensory quality of white bread, and analyse the dose-effect relationship.

5. Selection of study population

Subjects for the study will be aged between 18 and 40 inclusive with no known medical condition and otherwise must fulfil the inclusion and exclusion criteria.

① Inclusion criteria

Participants must be healthy males or non-pregnant females, non-lactating and aged 18 to 60 years old.

Participants with a BMI between 18.5 and 24.0 kg/m^2.

Able to tolerate fasting for at least 10 hours

②Exclusion criteria

Participants with impaired glucose tolerance or known history of diabetes mellitus or the use of antihyperglycemic drugs or insulin to treat diabetes and related conditions.

Suffer from any chronic diseases, such as AIDS, hepatitis, renal or heart disease or any other serious complications that may interfere with glucose metabolism.

Participants using any medication (eg steroids, protease inhibitors or antipsychotics, oral contraceptives, acetylsalicylic acid, etc.) or certain nutrition supplements that would interfere with the nutrient digestion, absorption, or glucose tolerance.

Participants having gastrointestinal diseases that may interfere with nutrient absorption, distribution, metabolism, excretion, intolerance to the study products and have known food allergies.

A major medical or surgical event requiring hospitalisation within the preceding 3 months.

6. Sample size estimation

The protocol of ISO 26642:2010 indicated 10 healthy subjects would be selected for a food GI determination, with the assumption of a dropout rate of 20%, at least 12 subjects were required to ensure a data-set of 10 subjects at each GI test.

7. Measurement index

The glucose values will be plotted against time and the Incremental Area Under Curve (iAUC) will be determined. GI will be calculated by expressing each participant's glucose iAUC for the test product as a percentage of the same participant's average iAUC for the reference food, as illustrated in the formula below.

$$GI = \left(\frac{\text{iAUC of test product}}{\text{Average iAUC of reference food}}\right) * 100$$

8. Research scheme

This study is exploratory. The specific research design is shown in the figure below.

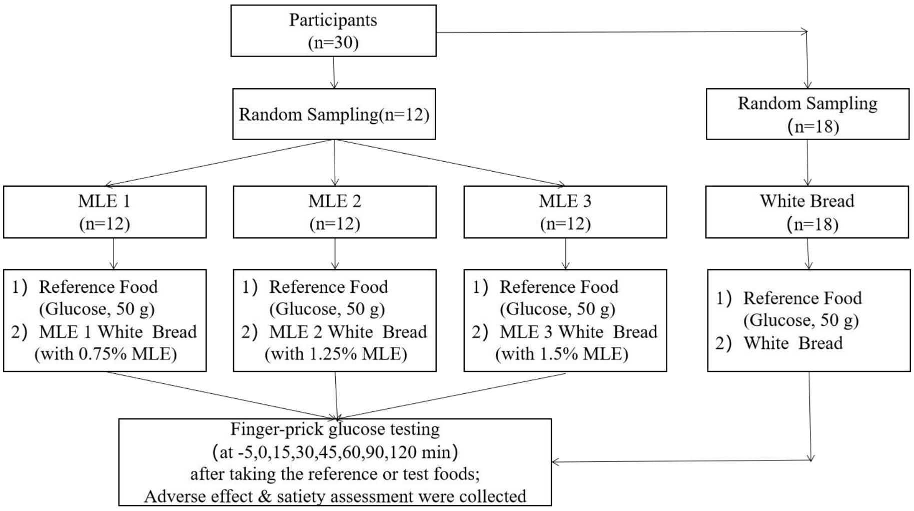

Twelve subjects were randomly selected from 30 subjects to participate the GI tests of the experiment groups, and 18 subjects participated in the test of control group. The investigator followed the specified randomization procedures of the protocol and ensured that the randomization code is broken only in accordance with the requirements of the protocol.

9. Ethics

The ethical approval for the study was obtained at the Chinese Academy of Medical Sciences and Beijing Union Medical College Hospital Ethics Review Committee (protocol code: HS-1763, first issued: November 27, 2018, latest updated on September 27, 2021).

10. Standard operation process

The test was carried out in strict accordance with ISO 26642:2010 standard. In this experiment, blood was taken from the fingertip of a disposable blood sampling needle.

[Each GI test cycle]

①Set up participant's testing area with labels, test record sheet, alcohol swabs, lancets, finger prick device and timer.

②Record weight of each participant with shoes off upon arrival for the test each time.

③Fill out top section of blood glucose test record and double check for accuracy.

④Label tube with -5, 0 labels containing participant's initials, date and test food. The participants' identity will be subsequently decoded for sample analysis.

⑤Ensure participant is using own personalised blood-taking supplies and is familiar with safety procedures as outlined in consent form.

⑥Wipe participants finger with alcohol pad, then prick finger for fasting samples. Note: The capillary blood samples will be collected by trained research staff or phlebotomist.

⑦Collect 350 μL of blood in test tube and mix blood with small amount of anti-coagulant at the bottom of the tube by rolling between hands. If glucose strips are used, blood is directly dropped on the strip and the values read immediately.

⑧Then the second fasting sample is taken at 0 minute and blood is collected accordingly. Taking 2 samples within 5 minutes is acceptable.

⑨Serve reference food/test food to the participant according to the study protocol with 250 mL of water

⑩Start timer with the first bite of test meal. Timers should be set at count down or count up to 2 hours.

⑪Make sure participant consumes test food or reference food and water within 5 to 10 minutes. However, if the food cannot be consumed in 10 minutes, the participant should try to consume the entire food, if possible, even if it takes longer than 10 minutes. Take as long as required to consume the entire test food or reference food. Participant may have to stop eating to take the 10 minute finger prick and continue eating.

⑫Record time taken to eat entire meal on the test record sheet.

⑬Prick finger, collect blood, roll tube, label tube, place in fridge. Continue this step for 30, 45, 60, 90, and 120 minutes, ensuring all tubes are properly labeled. If glucose strips are used, no need to collect blood, but can be analysed immediately.

⑭Used needles are thrown into the biohazard collection container. Any other contaminated waste is also disposed of in the appropriate container.

⑮Ensure test record is complete and accurate.

⑯Direct participants to snack area if they want a snack before leaving.

⑰Disinfect tables and kitchen counters with 70% ethanol solution.

⑱Participants will be reimbursed for their time participating in the study. If a participant withdraws from the study or becomes ineligible and are withdrawn by the investigator prior to completing the full study, they will be compensated for portions of the study completed to that point. If this occurs, the participant will be replaced in order to maintain a minimum of twelve (12) participants with complete data for statistical validity. The randomization sequence for the replacement will be the same as the participant who drops out from the study.

Note: A wash out period of three (3) days (72 hours) is required between two different tests.

11. Statistical analysis methods

All statistical analyses were performed using SAS version 9.4. The blood glucose data were calculated from the point values of all the time spots of every participant, and the means and standard deviation (SD) were calculated for the normality test. All data were processed and compared using origin 2021 and Graph Prism 7.0. Data were analyzed by one-way ANOVA and paired t-test and differences with values of $p<0.01$ or $p<0.05$ were considered significantly differences.

①Blood glucose measurement

Blood glucose level analysis instrument: Beckman Coulter AU480 automatic spectrophotometric analyzer with internal controls (CV<3.6%).

Blood collection: fingertip-prick blood sample collection.

②Calculation of GI

The GI was calculated with the reference glucose whose IAUC set as 100 (white bread is set as 71)，the GI value of the product is:

$$I_{mean}=\text{Test food IAUC/glucose IAUC}\times 100$$

③Satiety evaluation

The VAS scale was used for the comparison of satiety. Subjects' satiety change were record at 8 time points (0 min, 15 min, 30 min, 45 min, 60 min, 90 min and 120 min) within 2 hours after eating, and the data were collected and processed.

12. Publishing plan

This manuscript is planned to be submitted and published in 2023.
